# SHAPING LIVING IN SOLIDARITY AMONG OLDER VULNERABLE PEOPLE IN BRUSSELS

**DOI:** 10.1093/geroni/igz038.2140

**Published:** 2019-11-08

**Authors:** An-Sofie Smetcoren, Liesbeth De Donder

**Affiliations:** 1 Vrije Universiteit Brussel, Brussels, Belgium; 2 Vrije Universiteit Brussel, Brussels, Brussels Hoofdstedelijk Gewest, Belgium

## Abstract

This research explores how older people construct their view on ‘living in solidarity’. The data was collected during a cohousing project (from construction till occupation). Co-creation sessions with residents and project coordinators were analyzed. 7 conditions and success factors were unraveled that deemed important to realize ‘solidary housing’: 1) The challenge to unite individual and collective needs; 2) Continuous task to engage (candidate-) residents, from early beginning; 3) A targeted selection of residents; 4) Maximizing the competences of older people, 5) Developing a group identity consciousness, 6) Involving experts providing support for group- and individual trajectories, 7) Creating a climate of confidence. Solidary housing appeared to be a nuanced and layered concept which should not be overidealized. Participants demonstrate what is realistic, and how it can be shaped in daily practice. By discussing and deciding on group issues, the project coordinators created a shared platform for implicit and explicit agreements.

